# Feasibility and Reliability of an Automated Muscle Segmentation Pipeline Linking Thoracic Supine Kyphosis and Trunk Muscle–Fat% on CT

**DOI:** 10.3390/tomography12050059

**Published:** 2026-04-23

**Authors:** Tianxi Liang, Rian Atri, Sarah Joseph, Yiyuan Shao, Zhitong Zou, Adrian J. Villanueva, Aida Y. Prince, Renke Deng, Kurt Teichman, Xinzi He, Martin R. Prince

**Affiliations:** 1 Department of Radiology, Weill Cornell Medicine, New York, NY 10065, USA; til4023@med.cornell.edu (T.L.); hello@rian.fyi (R.A.); saj4023@med.cornell.edu (S.J.); ys3993@columbia.edu (Y.S.); amv4012@qatar-med.cornell.edu (A.J.V.); kut2002@med.cornell.edu (K.T.); xih4004@med.cornell.edu (X.H.); 2 Independent Researcher, Zhitong Zou, Brighton, BN2 1AD, UK; lancy5672004@gmail.com; 3 Independent Researcher, Aida Y. Prince, Brighton BN2 1AD, UK; aidap202327@gmail.com; 4 Graduate School of Arts and Sciences, Georgetown University, Washington, DC 20057, USA; rdeng2011@icloud.com; 5 Department of Radiology, Columbia University Vagelos College of Physicians and Surgeons, New York, NY 10032, USA

**Keywords:** thoracic supine kyphosis, Cobb angle, computed tomography (CT), fatty atrophy, muscle–fat%, deep learning, nnU-Net, muscle segmentation, intraclass correlation (ICC)

## Abstract

**Background:** As muscles atrophy, myocytes are replaced by fat and muscle strength diminishes, increasing thoracic supine kyphosis. Here, we investigate the relationship between muscle fat percentage (muscle–fat%) and thoracic supine kyphosis on CT. **Methods:** Thoracic Cobb angle was measured on supine CT scans from the AtlasDataset by four observers (n=533). Nine muscles were manually labeled on 100 scans (manual cohort). An nnU-Net model was trained on 80 cases with internal validation on 20 cases, then applied to segment the remaining 433 AtlasDataset scans (automated cohort). External segmentation benchmarking was performed on 30 cases from a separate open-source dataset. Associations between supine thoracic curvature and muscle–fat% were evaluated only in AtlasDataset. **Results:** Manual supine thoracic Cobb angle measurements demonstrated good inter-observer reproducibility (ICC(2,k) = 0.98) with a mean across-rater per-case SD of 3.4°. The nnU-Net achieved Dice scores >0.93 across all nine muscle groups on internal and external segmentation benchmarking. For both manual and automated cohorts, thoracic supine kyphosis correlated with muscle–fat% in the paraspinal (r = 0.35, 0.42), quadratus lumborum (r = 0.29, 0.33), vastus (r = 0.38, 0.32), psoas (r = 0.21, 0.23) and latissimus dorsi (r = 0.21, 0.17) muscles. **Conclusions:** Automated measurement of trunk muscle–fat% provides a reproducible imaging biomarker correlated with thoracic supine kyphosis on CT. Identifying fatty atrophy of core muscles may help identify potential targets for interventions in hyperkyphotic patients.

## Simple Summary

Thoracic kyphosis and muscle degeneration often occur together, but manual spine measurements on computed tomography scans are time-consuming and difficult to apply at scale. In this study, we developed an automated deep learning pipeline to segment trunk muscles and measure the percentage of fat within muscle on routine scans. Greater fat infiltration in several trunk muscles was associated with greater thoracic supine kyphosis, and the automated method showed strong reproducibility. These findings suggest that automated muscle analysis from existing scans may help quantify musculoskeletal decline and complement assessment of spinal curvature.

## 1. Introduction

**Figure A1:**
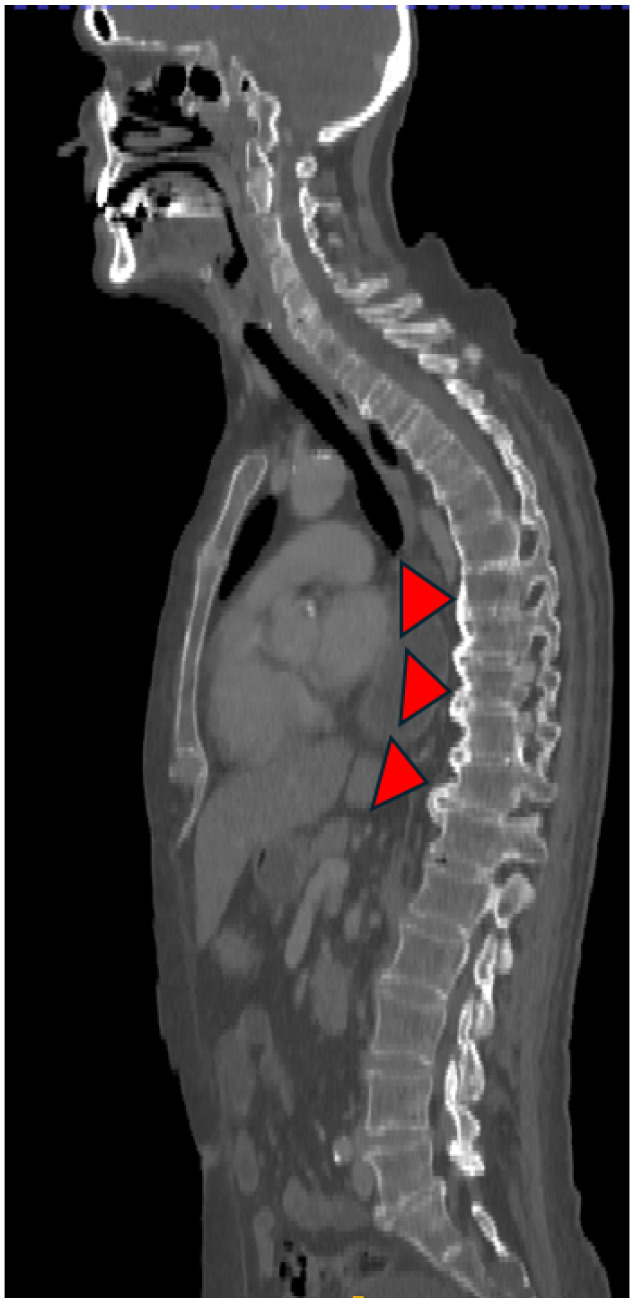
Sagittal midline CT reconstruction demonstrating fixed thoracic supine hyperkyphosis. Ossification of the anterior longitudinal ligament (red arrowheads) results in a rigid thoracic curvature that does not straighten despite supine positioning on the CT table, indicating structural rather than postural supine kyphosis.

Thoracic kyphosis, quantified by the Cobb angle on standing lateral X-rays [1], is associated with impaired mobility, pulmonary restriction, pain, and increased vertebral fracture risk in older adults [2]. Accurate measurement of the Cobb angle, however, can be challenging [3,4,5]. In younger, flexible spines, standing radiographs may yield a wide range of values depending on patient positioning [6]. A diurnal variation has been reported [6], with standing kyphosis increasing by approximately 5° between same-day morning and evening radiographs, presumably due to fatigue. Interestingly, there is minimal difference in Cobb angles between standing and supine positioning in older patients [7], and CT performed with supine positioning has been used for Cobb angle measurements [2]. Although a normal spine may straighten in the supine position, a spine habituated to thoracic kyphosis may not fully straighten even when lying supine; see Figure A1, which shows ossification of the anterior longitudinal ligament preventing dorsiflexion of the thoracic spine. Thus, supine CT may have less postural bias compared to standing X-rays for assessing the degree to which a patient can relax into normal spinal alignment [2,7]. Although manual Cobb angle measurements on CT can be performed with good reproducibility following appropriate training, it remains labor-intensive and reader-dependent, and is therefore underutilized in routine clinical practice [3,4,5]. Automated or surrogate biomarkers could improve throughput and enable opportunistic screening of sagittal deformity and identification of patients who may benefit from intervention.

Muscle composition analysis on CT offers a quantitative framework for assessing musculoskeletal health [2,8,9,10]. CT can quantify both muscle volume and intramuscular fat [2,9,10]. Intramuscular fat develops through both lipid accumulation within myocytes and expansion of intramuscular adipocytes [8]. Sarcopenia, the age-related loss of skeletal muscle mass and function, is associated with progressive reductions in muscle quantity and deterioration in muscle quality through fatty infiltration [8,11]. Muscle mass has been reported to decline by approximately 3% to 8% per decade after the age of 30, with faster decline at older ages [11].

In this study, muscle–fat% is defined as the percentage of voxels within each segmented muscle with attenuation below −20 HU, and is used as a quantitative marker of intramuscular fatty atrophy and muscle decline with aging, inactivity, or chronic spine pathology [2,10,12].

Elevated muscle–fat% in paraspinal and trunk muscles has been associated with reduced strength, imbalance, and deformity progression in lumbar and cervical regions [10,12,13,14,15,16,17]. However, the relationship between CT-derived muscle–fat% of thoracic or trunk musculature and thoracic kyphosis in supine position remains incompletely characterized. Establishing this link could clarify how muscular degeneration with fatty atrophy contributes to structural curvature and functional impairment.

Building on these insights, we propose an automated, reproducible pipeline for thoracic alignment analysis that integrates manual and deep learning-based muscle segmentation with voxelwise quantification of muscle–fat% [2,18]. Muscle volume is directly measured from the segmentation volumes. Muscle–fat% is measured by counting intramuscular voxels with attenuation below −20 HU [2,10].

Using the AtlasDataset [19,20], we first validated inter-observer variability of manual Cobb angle measurements [21] from the CT scans. Cobb angle measurements from the multiple observers were then averaged to create the reference standard for the supine thoracic kyphosis. Then we trained an nnU-Net segmentation model [18] to segment and compute muscle–fat% across nine bilateral muscle groups in the entire 533-case CT cohort. The segmentation model was validated in another CT dataset [22]. We next tested correlations between muscle–fat% and supine thoracic curvature to determine whether muscle fatty atrophy is associated with increased supine kyphotic deformity. Our overarching aim is to evaluate muscle–fat% as a scalable surrogate of spinal curvature—providing quantitative, opportunistic assessment of supine kyphosis on routine CT scans [2].

This integrated framework supports reproducible measurement of spinal alignment and muscle composition, bridging radiological and biomechanical markers of sagittal imbalance while laying groundwork for population-scale, AI-assisted spine health analytics.

## 2. Materials and Methods

**Figure 1:**
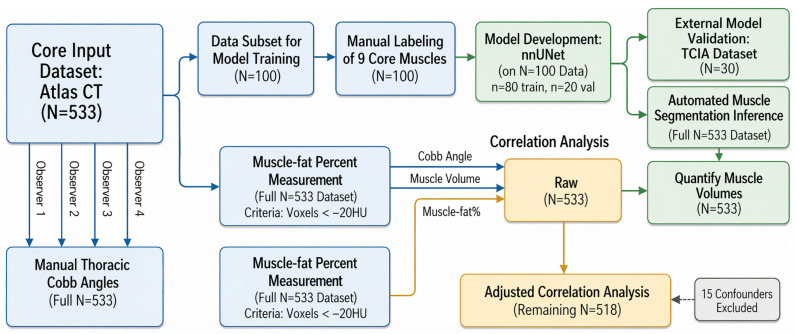
Overall study pipeline for automated muscle segmentation and thoracic supine kyphosis analysis on CT. Whole-body CT scans from the AtlasDataset (n=533) supported two parallel study components: manual thoracic supine Cobb angle measurement and development of an automated muscle segmentation pipeline. For Cobb analysis, all 533 scans were measured manually, with four observers contributing to reliability assessment. In parallel, 100 AtlasDataset scans were manually labeled in ITK-SNAP for nine bilateral muscle groups and used for nnU-Net development, with an 80-case training split and a 20-case internal validation split. External validation was performed on a TCIA cohort (n=30). The trained nnU-Net was then applied to the remaining 433 AtlasDataset scans to generate automated muscle segmentations, from which muscle volume and intramuscular fat percentage (muscle–fat%) were quantified using a threshold of HU <−20. Correlation analysis integrated thoracic supine Cobb angle, muscle volume, and muscle–fat%. Initial analysis included all AtlasDataset cases (n=533), after which 15 predefined confounded cases were excluded, yielding an adjusted cohort of n=518. In the manually labeled subset (n=100), correlations used manual muscle masks, whereas the remaining scans formed the automated cohort (n=433) for large-scale analysis using nnU-Net segmentations.

An overview of the full study workflow is shown in Figure 1, including AtlasDataset case selection, manual thoracic supine Cobb angle measurement, manual muscle segmentation for nnU-Net development, external segmentation benchmarking, automated muscle–fat% quantification, confounder exclusion, and downstream correlation analysis between muscle–fat% and thoracic supine kyphosis.

### 2.1. Dataset and Manual Thoracic Supine Kyphosis on CT

We utilized de-identified adult whole-body CT volumes from the AtlasDataset [19,20], a publicly available resource providing high-resolution, label-rich imaging suitable for anatomical measurement and deep learning-based model development. A separate external cohort of CT scans was obtained from The Cancer Imaging Archive (TCIA) CT-ORG collection for independent segmentation performance evaluation [22]. For external segmentation benchmarking, trunk muscles were manually segmented on a subset of the external cohort (n=30) using the same label taxonomy and protocol as AtlasDataset, enabling Dice-based performance evaluation.

**Figure 2:**
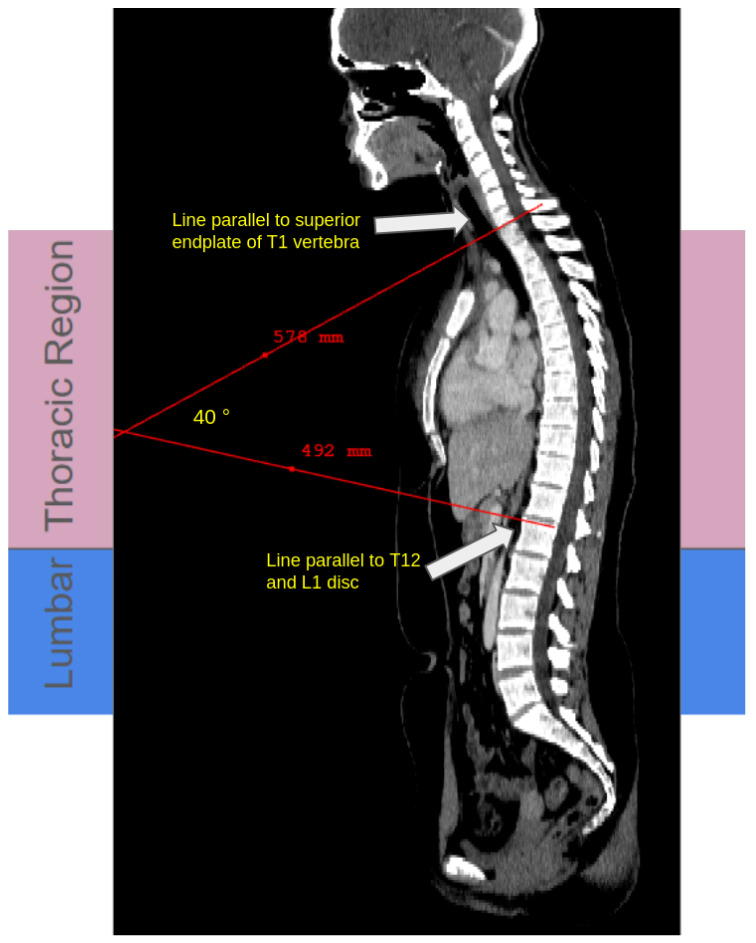
Manual thoracic supine kyphosis measurement on CT. Representative mid-sagittal CT reconstruction showing the thoracic and lumbar regions, with red lines drawn parallel to the superior endplate of T1 and the inferior endplate of T12/L1. The intersection of these red lines defines the supine Cobb angle, representing the degree of residual thoracic supine kyphosis with supine positioning [1]. Regional color overlays indicate the thoracic (pink) and lumbar (blue) segments. Measurements and visualization were performed using ITK-SNAP [23]. The same convention was applied across the full internal cohort (n=533) used for inter-observer reliability analysis.

Thoracic supine kyphosis was quantified using the Cobb angle measured on mid-sagittal CT reconstructions, defined between the superior endplate of T1 and the inferior endplate of T12/L1, following standard radiographic conventions (Figure 2) [1].

#### 2.1.1. ManualCobb Angle Measurement and Reliability

Supine thoracic Cobb angles for all cases included in this study were independently measured by four trained observers in all 533 cases. Inter-observer agreement was quantified using the two-way random-effects intraclass correlation coefficient (ICC(2,*k*)) with absolute agreement and bootstrap 95% confidence intervals [21]. For all subsequent analyses, the mean Cobb angle across the four observers was used as the reference value.

#### 2.1.2. nnU-Net Segmentation Benchmark

For segmentation model development and benchmarking, 100 manually labeled AtlasDataset scans were partitioned at the subject level into 80 cases for nnU-Net training and 20 cases for internal validation. An independent external segmentation benchmarking cohort (n=30) was used exclusively for segmentation performance evaluation. The internal validation set was used solely for monitoring convergence and model selection and was not used for final benchmarking.

#### 2.1.3. Muscle–Fat% Quantification and Correlation Analysis

For biomarker analysis, intramuscular fat percentage (muscle–fat%) was computed within muscle segmentations and correlated with supine thoracic Cobb angle using Pearson’s correlation. Correlations were computed in AtlasDataset only using two cohorts: (i) the manual cohort (n=100) with manual muscle segmentations, and (ii) the automated cohort (n=433) segmented using the trained nnU-Net model.

### 2.2. Confounder Identification and Exclusion

**Figure 3:**
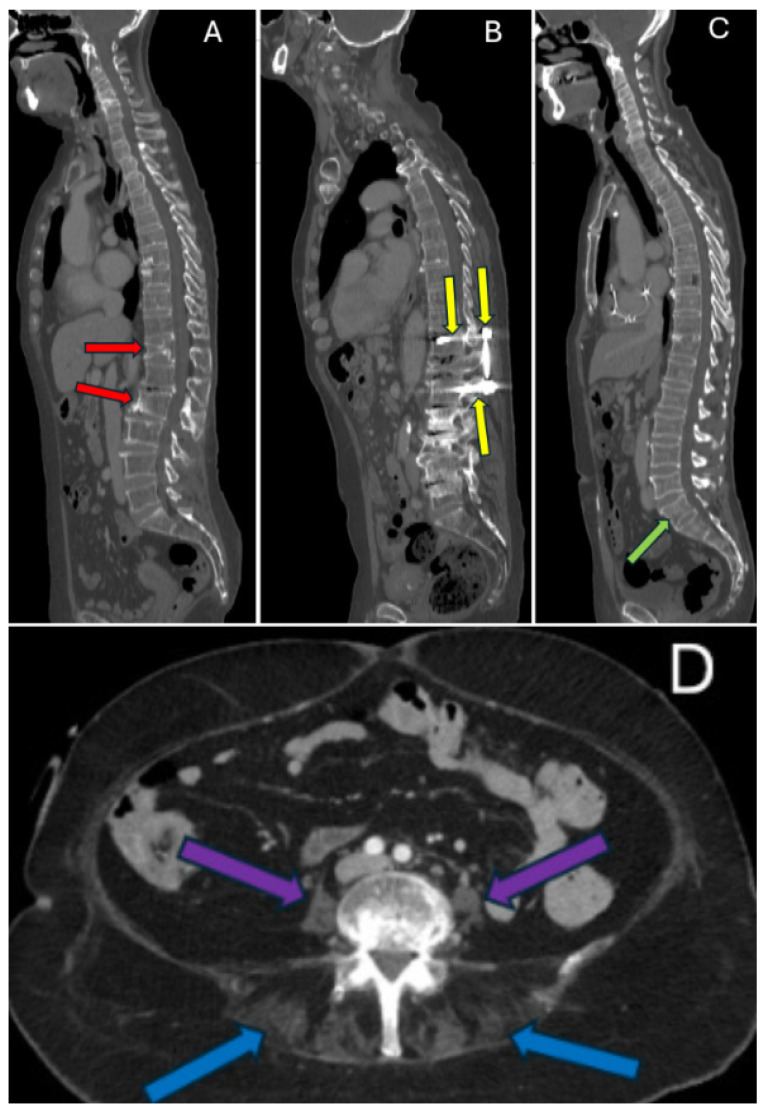
Examples of confounders that may affect spine Cobb angle. (**A**): Vertebral body compression fractures at T12 and L2 (red arrows). (**B**): Spine surgery spanning from T11 to L3 with metallic rod and screws (Yellow arrows). (**C**): transitional vertebra with partial sacralization of L5 (green arrow). (**D**): Profound fatty atrophy of paraspinal muscles (blue arrows); psoas (purple arrows) is not compatible with ambulation.

To assess robustness of associations between thoracic supine kyphosis and muscle–fat percentage, cases were screened for predefined confounders expected to disrupt the biomechanical relationship between upright posture and trunk muscle composition. These included prior spine surgery and profound fatty muscle atrophy (paraspinal muscle–fat% >50%), which was judged likely to reflect non-ambulatory status. Transitional vertebrae were evaluated separately in a sensitivity analysis rather than included in the main predefined exclusion set. Confounder assessment was performed by a board-certified radiologist with over 30 years of experience in musculoskeletal and spine imaging. Examples of these confounders are shown in Figure 3. Excluded case identifiers and quality-control removals are listed in Table A1.

### 2.3. Muscle Segmentation on CT

Nine bilateral trunk muscle groups (psoas, quadratus lumborum, paraspinal, latissimus dorsi, iliacus, rectus femoris, rhomboid, trapezius, and vastus) were manually segmented on 100 AtlasDataset CT scans using ITK-SNAP [23,24]. Manual reference labels were initially created by trained observers (Y.S., S.J., R.A., T.L., A.Y.P., A.J.V., and Z.Z.) and subsequently reviewed and refined by a board-certified radiologist (M.R.P.) with more than 30 years of experience.

**Figure 4:**
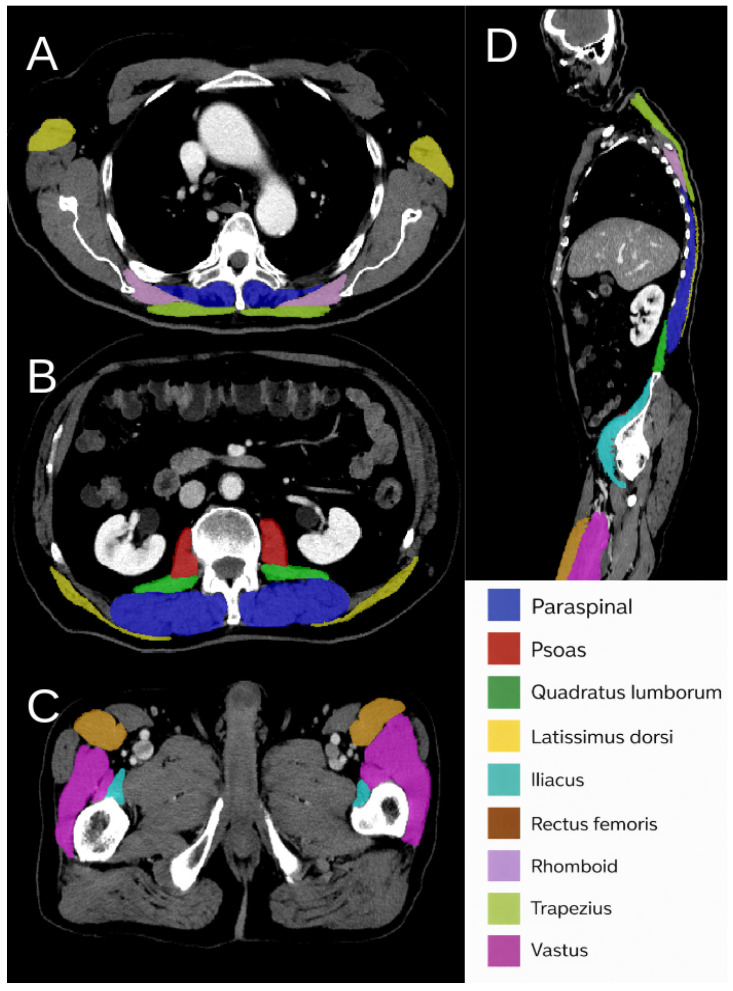
Manual muscle annotations used for supervision and QA (CT). (**A**–**C**): representative axial slices at thoracic, abdominal, pelvic levels, respectively, with color-coded overlays for nine bilateral muscle groups (paraspinal, psoas, quadratus lumborum, latissimus dorsi, iliacus, rectus femoris, rhomboid, trapezius, vastus). (**D**): mid-sagittal reconstruction showing the craniocaudal extent of the labels. Annotations were created in ITK-SNAP [23] and used to train a single-fold nnU-Net 3d_fullresmodel [18]; detailed training and validation results mentioned in Section 2.6.

The 100 manually segmented CT scans used for nnU-Net model development were divided 80:20 for model training and validation. These manual annotations served as ground truth for training a 3D nnU-Net [18], which was then applied to segment the remaining AtlasDataset inference cohort (n=433) for biomarker analyses, and the external TCIA cohort used for segmentation benchmarking. Representative manual annotations illustrating the muscle label taxonomy and craniocaudal distribution are shown in Figure 4.

Preprocessing included isotropic resampling, intensity clipping to a soft-tissue Hounsfield unit range, and z-score normalization. A fixed training–validation split was used for model selection, with the external cohort reserved exclusively for final segmentation benchmarking. The detailed training configuration (loss functions, augmentations, learning schedule) and quantitative validation metrics are reported in Section 2.6.

### 2.4. Muscle–Fat% Quantification on CT

Following automated or manual muscle segmentation, voxel-level intensity filtering was applied to quantify intramuscular fat content. Muscle masks were generated using the nnU-Net model [18], and voxel Hounsfield units (HU) were extracted within each segmented muscle. Voxels with HU values below −20 were classified as fat-containing tissue, based on established CT attenuation thresholds for intramuscular adiposity and muscle quality assessment [2,9,10]. Intramuscular fat fraction (muscle–fat%) was then computed as the ratio of low-attenuation voxels (HU <−20) to the total number of voxels within each 3D muscle mask.

For the quality-control comparison on development scans, intramuscular fat fraction (muscle–fat%) was computed twice per muscle per subject—once using the manual mask and once using the automated mask from the frozen model—yielding paired measurements for mean ± SD summaries and tables reported in Section 3.

**Figure 5:**
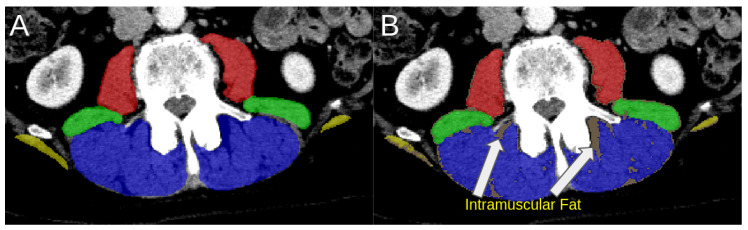
Voxel-based muscle–fat% filtering on CT. (Case 102) (**A**): representative axial slice showing normal trunk muscle morphology across segmented muscle groups (paraspinal in blue, quadratus lumborum in green, psoas in red, and latissimus dorsi in yellow). (**B**): corresponding slice with intramuscular fat labeled, showing low-attenuation voxels (Hounsfield unit < −20) in light brown. In this example case, the paraspinal, quadratus lumborum, psoas, and latissimus dorsi muscles exhibit fat fractions of 12%, 12%, 5.7%, and 22%, respectively. These values are computed from the full 3D muscle volumes rather than the single axial slice shown here.

Figure 5 illustrates the voxel-based filtering process and its effect on differentiating healthy muscle from fatty atrophy.

### 2.5. Statistical Analysis

The primary endpoint was the association between thoracic supine kyphosis, quantified by the Cobb angle on the sagittal plane, and muscle–fat% derived from CT. All analyses were conducted in Python 3.10.12 using scipy.statsand pingouin [25], with visualizations generated in matplotlib.

Thoracic supine kyphosis measurements for all internal cases were performed independently by four trained observers. Inter-observer consistency was quantified using the two-way random-effects intraclass correlation coefficient ICC(2,*k*) with absolute agreement and bootstrap 95% confidence intervals [21]. The mean Cobb angle across observers was used in all subsequent analyses. Detailed four-observer reliability summaries are reported in Table A2.

For segmentation model development, nnU-Net training and validation were performed using fixed AtlasDataset partitions (n=80 for training and n=20 for validation). These cohorts were used exclusively for model optimization and were not used for segmentation benchmarking.

For biomarker analysis, Pearson’s correlation coefficient (*r*) was used to assess the relationship between thoracic supine Cobb angle and muscle–fat%, with two-sided *p*-values reported. Correlations were computed in AtlasDataset in two cohorts: (i) the manual cohort (n=100), and (ii) the automated cohort (n=433). Baseline correlations were computed on the full cohorts. Because correlations were evaluated across nine muscle groups, *p*-values are interpreted cautiously as exploratory.

Sex-stratified correlations are reported in Table A3. Additional sensitivity analyses for vertebral compression fractures and transitional vertebrae are reported in Table A4 and Table A5.

The external cohort was used exclusively for segmentation performance verification and was not included in any muscle–fat% or supine kyphosis correlation analyses due to inadequate dataset information.

### 2.6. Model and Training

A 3D nnU-Net 3d_fullres configuration [18] was trained for automated trunk muscle segmentation using a compound Dice+cross-entropy loss function. The total loss $L_{\mathrm{total}}$ combined the soft Dice loss $L_{\mathrm{Dice}}$ and voxel-wise cross-entropy loss $L_{\mathrm{CE}}$ as:

(1) $$L_{\mathrm{total}}=L_{\mathrm{Dice}}+L_{\mathrm{CE}},$$

where the Dice term was defined as:

(2) $$L_{\mathrm{Dice}}=1-\frac{2\sum_{c}p_{c}g_{c}+\epsilon }{\sum_{c}p_{c}^{2}+\sum_{c}g_{c}^{2}+\epsilon },$$

with $p_{c}$ and $g_{c}$ denoting the predicted and ground-truth probabilities for class *c*, respectively, and ϵ a small constant for numerical stability. The cross-entropy term penalized voxel-wise misclassification:

(3) $$L_{\mathrm{CE}}=-\sum_{c}g_{c}\log(p_{c}).$$

The model was optimized using the AdamW optimizer with a cosine annealing learning rate schedule (initial $\eta_{0}=1\times 10^{-3}$, minimum $\eta_{\min}=1\times 10^{-6}$). A batch size of 2 and gradient accumulation of 4 steps were employed to fit the 3D context window (patch size $128^{3}$) within GPU memory constraints. Weight decay ($1\times 10^{-5}$) and exponential moving average (EMA) parameter tracking (α=0.99) were used to stabilize training.

Standard nnU-Net data augmentations, including random rotations, elastic deformations, gamma correction, mirroring, and intensity jitter, were applied on-the-fly. We used nnU-Net’s CT preprocessing, with volumes resampled to 2.5 × 0.871 × 0.871 mm (z,y,x) using cubic interpolation in-plane and nearest along z for images. Intensities were clipped to −95 to +116 HU based on dataset foreground percentiles (0.5th–99.5th), and then z-score-normalized per case. Specific preprocessing information can be found at the publicly available code repository.

**Figure 6:**
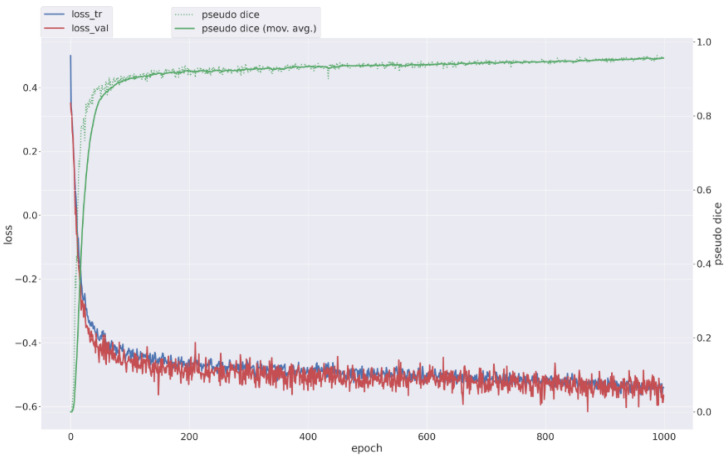
nnU-Nettraining performance on CT. Training and validation loss trajectories with corresponding EMA pseudo-Dice values across epochs, showing stable convergence of the nnU-Net 3d_fullres configuration.

Model convergence was monitored through pseudo-Dice and EMA validation metrics across epochs. Training and validation trajectories are shown in Figure 6, demonstrating stable optimization and consistent convergence behavior.

### 2.7. Computational Environment

Model training and inference were conducted on a Linux (x86_64) workstation equipped with an NVIDIA GeForce RTX 4090 GPU (24 GB GDDR6X memory). Key software dependencies and hardware specifications are summarized in Table 1 to ensure reproducibility.

## 3. Results

### 3.1. Cohort Characteristics and Potential Confounders

Although AtlasDataset did not include demographic metadata, we inferred apparent sex categories (male/female) from external anatomy on CT (302 males, 231 females). During radiologic review, several anatomical and clinical factors were identified that were judged to confound analysis of the relationship between muscle fatty atrophy and thoracic supine kyphosis. Evidence of prior spine surgery was present in 10 patients, and vertebral compression fractures were identified in 52 patients. Five had profound fatty atrophy of most muscles including paraspinal muscles exceeding 50% muscle–fat%. These patients were likely not ambulatory and thus their spines were not subject to the normal forces occurring with upright posture. Twenty-five patients had transitional vertebrae at the L5–S1 level, making identification of vertebral levels for thoracic Cobb angle measurement more challenging.

Cases with prior spine surgery or profound fatty atrophy (paraspinal muscle–fat% >50%) were excluded from analyses evaluating associations between thoracic supine kyphosis and muscle–fat%. For the evaluation of the trapezius muscle, an additional case was excluded because the CT reconstruction cropped part of one trapezius muscle laterally, another case was excluded due to trapezius muscle absence on one side, and two further cases with thoracic surgery possibly affecting trapezius were also excluded. Vertebral compression fractures and transitional vertebrae were evaluated in further sensitivity analyses (Table A4 and Table A5), and excluded case identifiers are provided in Table A1.

### 3.2. Segmentation Performance by Muscle Group (CT)

The trained nnU-Net model achieved strong segmentation performance across all nine bilateral muscle groups. Mean Dice coefficients ranged from 0.962 to 0.988 on the training cohort, 0.948 to 0.976 on the internal validation cohort, and 0.932 to 0.965 on the external segmentation benchmark cohort (Table 2). This modest Dice reduction from training to internal validation to external segmentation benchmarking cohorts is consistent with expected generalization effects under cohort shift. Accuracy of the segmentations was further verified by visual inspection of a random 5% subset of cases when the model was applied to the remaining 433 Atlas cases for automated cohort analysis.

Segmentation performance by muscle group and cohort is summarized in Table 2. Dice values correspond to the cohort-specific mean Dice coefficient computed per muscle.

### 3.3. Thoracic Supine Kyphosis Reliability Across Four Observers

Inter-observer agreement for manual thoracic supine kyphosis (Cobb angle on the midline sagittal reconstruction) was assessed across four trained readers using the two-way random-effects intraclass correlation coefficient with absolute agreement (ICC(2,*k*)) [21]. Reliability was evaluated on the internal cohort (n=533 cases). The resulting ICC(2,*k*) was 0.98 (bootstrap 95% CI: 0.977–0.982), indicating *good* inter-observer agreement. Although the Cobb angle itself can be measured in about 2 min per case, the total workflow time—including launching ITK-SNAP and loading the CT volume—averaged approximately 5 min per case. In contrast, muscle–fat% quantification is fully automated once segmentations are generated, requiring no per-case operator time.

**Figure 7:**
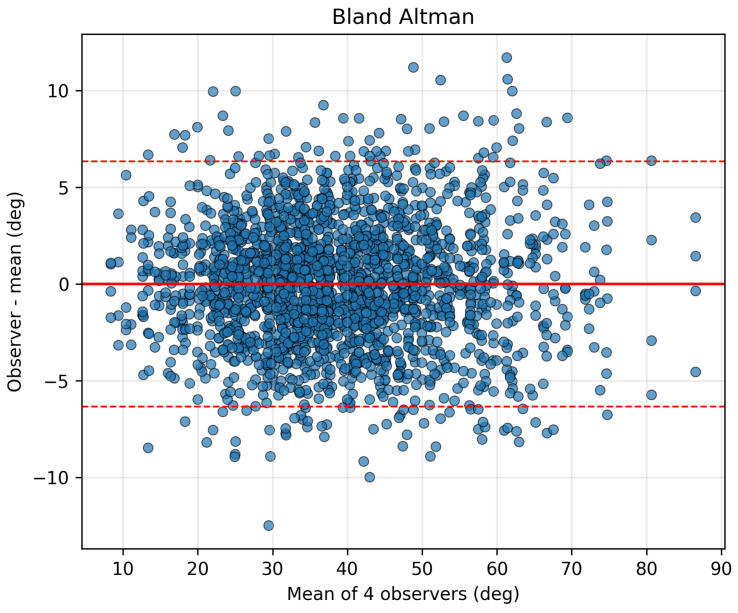
Inter-observer agreement for thoracic supine kyphosis measurements on CT (internal cohort). Bland–Altman plot [26] showing the difference between each observer’s measurement and the four-observer mean plotted against the mean thoracic Cobb angle across the internal cohort (n=533). The solid red line indicates the mean bias, and the dashed red lines indicate the 95% limits of agreement. The corresponding ICC(2,*k*) was 0.98 (bootstrap 95% CI: 0.977–0.982).

Mean thoracic supine Cobb angles were consistent across readers (Rater 1: 39.4 ± 13.9°, Rater 2: 38.3 ± 13.3°, Rater 3: 38.0 ± 12.8°, Rater 4: 38.6 ± 14.0°), with a grand mean of 38.6° across all ratings. The mean across-rater per-case standard deviation was 3.4 ± 1.6°, indicating that a typical single observer’s measurement deviated from the four-observer mean by a few degrees. Occasional larger deviations were observed, as visualized in the Bland–Altman plot (Figure 7) [26], which shows the distribution of observer differences and corresponding 95% limits of agreement.

### 3.4. Association Between Thoracic Supine Kyphosis and Muscle–Fat%

Mean muscle–fat% ranged from a low of 2.5% in the rectus femoris to higher levels of 15% (paraspinal), 14% (quadratus lumborum) and 19% (latissimus dorsi); see Table 3. We first computed baseline Pearson correlations (*r*) and *p*-values between thoracic supine kyphosis (Cobb angle) and intramuscular fat percentage for the full AtlasDataset cohorts—the manual cohort (n=100) and the automated cohort (n=433)—as shown in Table 4 (first and third columns). We then performed sensitivity analyses excluding cases with prior spine surgery and cases with profound fatty atrophy (paraspinal muscle–fat% >50%), yielding effective sample sizes of n=97 (manual cohort) and n=421 (automated cohort), Table 4 (second and fourth columns). The following Atlas cases were removed from manual cohort correlations: {101, 103, 155}, and from the automated cohort: {74, 94, 98, 237, 252, 288, 303, 387, 477, 480, 503, 531}. Trapezius analyses required additional removals due to missing trapezius labels or chest surgery: {76, 274, 275, 396}. These exclusions apply only to cases present in each analysis cohort; several confounders overlap, and not all flagged cases occur in the manually segmented subset.

Additional sensitivity analyses evaluating vertebral compression fractures and transitional vertebrae are provided in the appendix (Table A4 and Table A5). Across analyses, correlation patterns remained anatomically consistent, with the strongest associations observed in posterior trunk musculature.

Correlation analyses were first computed on the full AtlasDataset cohorts (manual cohort n=100; automated cohort n=433). In predefined sensitivity analyses, cases with prior spine surgery or profound fatty atrophy were excluded, yielding effective sample sizes of n=97 (manual cohort) and n=421 (automated cohort). Excluded case identifiers are reported in Appendix A. Pearson correlation results computed prior to exclusion, as well as results after exclusions and sex-stratified analyses, are reported in Section 3 and Appendix A.

#### Visualization of Correlations

**Figure 8:**
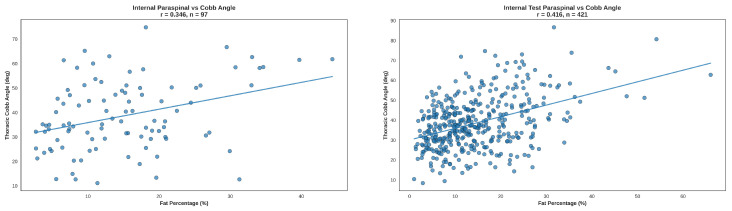
Paraspinal muscle–fat% vs. thoracic supine Cobb angle after confounder exclusion and quality control. (**Left**): manual cohort with manual muscle segmentations (n=97). (**Right**): automated cohort with nnU-Net segmentations (n=421). Plots reflect datasets after exclusion of cases with prior spine surgery, profound fatty atrophy (paraspinal muscle–fat% >50%), and a small number of additional quality-control removals due to segmentation artifacts.

**Figure A2:**
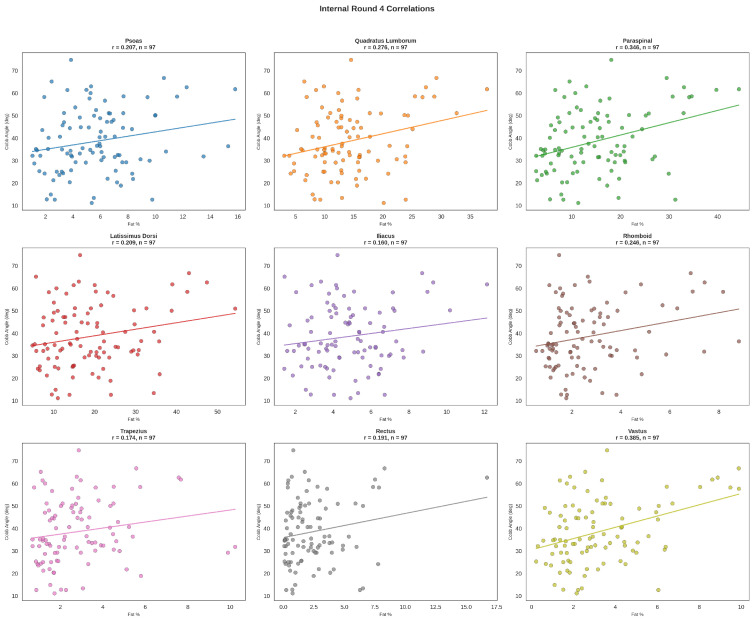
Manual-segmentation cohort (raw n=100; adjusted n=97): thoracic Cobb angle vs. muscle–fat% for all nine muscles (3 × 3 grid). The adjusted set excludes the adjusted-analysis exclusions listed in Table A1. Each subplot displays Pearson’s *r* and two-sided *p*-value.

**Figure A3:**
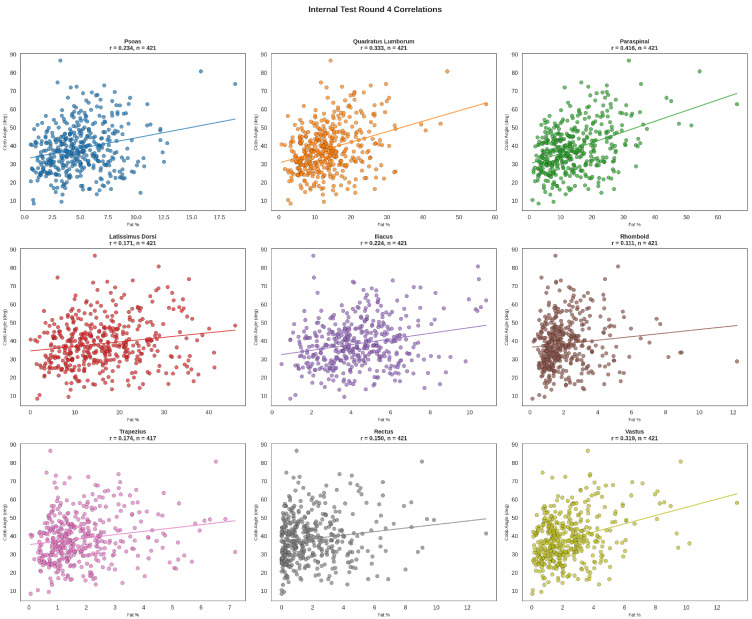
Automated nnU-Net cohort (raw n=433; adjusted n=421): thoracic Cobb angle vs. muscle–fat% for all nine muscles (3 × 3 grid). The adjusted set excludes the adjusted-analysis exclusions listed in Table A1. Each subplot displays Pearson’s *r* and two-sided *p*-value.

Figure 8 illustrates the association between thoracic supine kyphosis and paraspinal muscle–fat% as a representative example. Plots are shown for the manual cohort and the automated cohort after exclusion of cases with prior spine surgery, profound fatty atrophy, and a small number of additional cases removed during quality control due to segmentation artifacts. The resulting effective sample sizes for the paraspinal analysis were n=97 (manual cohort) and n=421 (automated cohort). Figure A2 provides the full 3 × 3 scatter-plot grid for all nine muscles in the manual cohort, and Figure A3 provides the corresponding grid for the automated cohort.

## 4. Discussion

### 4.1. Interpreting the Link Between Muscle–Fat% and Thoracic Curvature

Across both the manual and automated cohorts, higher thoracic supine kyphosis was consistently associated with greater intramuscular fat percentage, confirming a relationship between muscle fatty atrophy and increased thoracic supine kyphosis (Table 4; Figure 8; Figure A2 and Figure A3). This association was similar in males and females and did not appear to be materially affected by vertebral compression fractures or transitional vertebrae. Because this open-source dataset includes only images and no metadata, the effects of demographic variables not apparent on imaging could not be assessed; the potential influence of additional covariates (e.g., age, body mass index, physical activity level, or comorbidities) could not be evaluated.

The strongest relationship was found in the posterior thoracolumbar paraspinal extensor muscles, with weaker relationships in other muscles. This pattern mirrors the biomechanical hierarchy of postural control, with the paraspinal muscles, which are most responsible for counteracting anterior flexion, demonstrating the highest degree of fatty infiltration with increasing supine kyphosis. This convergence across datasets reinforces the biological plausibility of muscle–fat% as a muscle quality biomarker and aligns with prior literature linking paraspinal degeneration to sagittal imbalance and deformity progression [12,13,14,15,16,17] (see also Table 4). Our findings are also broadly consistent with systematic review evidence that paraspinal fatty infiltration is a recurrent feature across spine-related imaging studies, while extending that literature to automated CT-derived multi-muscle quantification in supine thoracic curvature assessment [27]. The fact that even thigh muscle–fat% in the vastus significantly correlated with thoracic supine kyphosis suggests that generalized fatty muscle atrophy is associated with increasing supine kyphosis [8,11].

The higher mean fat percentage observed in the paraspinal (15%) and quadratus lumborum (14%) muscles across all cases may reflect the fact that these muscles occupy anatomically constrained compartments defined in part by surrounding osseous structures, such that loss of contractile muscle tissue is preferentially replaced by fat. By contrast, muscles with greater freedom to decrease in size may show lower measured muscle–fat%, because loss of contractile tissue does not necessarily require volumetric replacement by fat. This may partly explain the lower mean values observed in the vastus (3.2%), rectus femoris (2.5%), trapezius, and rhomboid. Trapezius also had a high mean muscle–fat% which may reflect its complex, sheet-like anatomy, thin with a lot of surface which accentuates partial volume averaging incorporating surface fat into the segmentations. So it is not surprising that paraspinal and quadratus both had strong correlations. Strong vastus (a leg muscle not even touching the spine) muscle–fat% correlation with supine kyphosis suggests that generalized muscle atrophy throughout the body correlates with thoracic supine kyphosis.

### 4.2. Impact of Confounder Control and Sex Stratification

We evaluated whether predefined confounders materially influenced the observed associations between thoracic supine kyphosis and muscle–fat% by recomputing correlations before and after exclusion of prior spine surgery and profound fatty atrophy (paraspinal muscle–fat% >50%). Across both internal cohorts, excluding these cases yielded consistently higher correlation coefficients across all nine muscles (Table 4). This pattern supports the validity of the exclusion criteria: postoperative anatomy and extreme, likely non-ambulatory fatty degeneration plausibly disrupt the biomechanical relationship between trunk muscle quality and upright sagittal alignment, and their removal strengthens the underlying signal.

In contrast, stratification by sex showed only modest differences in effect size across muscles (Table A3). While some muscle groups demonstrated numerically higher or lower correlations in one sex, the overall pattern of association remained similar and did not indicate a systematic sex-driven confounding effect. These results suggest that sex is unlikely to be a dominant confounder for the supine kyphosis–muscle–fat% relationship in this dataset, although larger cohorts with complete demographic metadata would enable more definitive covariate-adjusted modeling. Sex-stratified correlations were directionally consistent and did not materially change interpretation; full sex-split results are reported in Table A3. Although transitional vertebrae at L5-S1 could confuse observers about the vertebral body levels, removing these cases with transitional vertebrae did not affect the results.

### 4.3. Cobb Angle Measurements

Cobb angle is prone to bias and is known to have limited reproducibility for single observers [3,4,5]. The mean across-rater per-case standard deviation of thoracic supine Cobb angle across the four observers was 3.4°, indicating that a typical single observer’s measurement differs from the four-observer mean by a few degrees. The Bland–Altman plot (Figure 7) [26] shows that 5% of cases are more than 6 degrees different from the mean of all observers. This supports the use of muscle–fat percent as a complementary and automatic method for evaluating hyperkyphosis risk on CT scans. Muscle–fat% also identifies targets for intervention to stimulate muscle strength and can precisely track progress in rebuilding muscle strength. Analogous muscle composition biomarkers may also be measurable on MRI, which could extend this framework to non-ionizing imaging modalities.

### 4.4. Pipeline Significance: Toward Automated Surrogate Biomarkers

The integration of reliable manual references, deep learning segmentation, and standardized HU-based fat quantification forms a reproducible end-to-end framework for assessing hyperkyphosis on routine CT. Unlike traditional Cobb measurements, which require reader input and geometric annotation, muscle–fat% can be extracted directly from volumetric data—automatically, reproducibly, and at scale, requiring only a few seconds of computer time. This transforms CT imaging from a qualitative tool into a quantitative platform for spinal health analytics (see segmentation performance in Table 2 and voxel-level filtering in Figure 5). Consistent lower muscle–fat% in the model segmentations compared to manual segmentation appears to reflect superior definition of muscle fat boundaries by the model compared to manual contours by the observers.

Physiologically, the relationship between muscle–fat% and supine kyphosis likely reflects a self-reinforcing cycle: progressive supine kyphosis from muscle weakness leads to decreased activity, promoting further muscle atrophy and fatty infiltration, which in turn reduces postural support and deepens curvature. Quantifying this cycle through a standardized muscle–fat% metric captures the mechanical consequences of spinal imbalance in a way that traditional Cobb angle measurements cannot. The ability to measure muscle–fat% automatically positions it as a scalable surrogate biomarker of sagittal supine curvature—one that complements, and in certain contexts may replace, explicit Cobb measurements for large-scale screening. This can be performed opportunistically on CT scans performed for other purposes as complementary information obtained at negligible additional cost.

### 4.5. Clinical and Research Implications

From a translational standpoint, automated muscle–fat% quantification has the potential to substantially enhance the impact and value of CT scanning. Potential benefits include opportunistic screening, monitoring, prognosis, precision rehabilitation, and population-scale analytics. Routine thoracoabdominal CT scans performed for a variety of indications could undergo automatic analysis of muscle–fat%, thereby providing additional information on musculoskeletal health that is not currently provided in routine radiology reporting. This assessment of muscle volumes and muscle–fat% is opportunistic because it does not require any additional radiation exposure or exam time for the patient. There is only a minimal, if any, increase in cost for running this automated algorithm, and the outputs could be readily quality-control-checked by radiologists when finalizing their reports.

In patients being assessed for thoracic kyphosis, back pain, and related back disorders, repeated muscle–fat% measurements over time, perhaps annually, could track muscular degeneration or recovery and serve as a quantitative indicator of core muscle health. Measuring the rate of change in muscle volume and muscle–fat% may also help predict future progression, thereby providing prognostic information.

Identifying specific muscles with high muscle–fat% may also create opportunities for therapeutic intervention. Targeting affected muscles or muscle groups with tailored strengthening and conditioning exercises could potentially counteract the observed degenerative pattern or slow its progression. Follow-up imaging could help guide therapeutic adjustments, and in some settings MRI could be used instead of CT to avoid additional radiation exposure.

The standardized nature of the pipeline also allows integration into large datasets for epidemiologic studies of posture, aging, and sarcopenia. Because these metrics can be derived simply and at low incremental cost, large-scale analyses may be practical and cost-effective.

By bridging anatomical, compositional, and geometric features, this pipeline facilitates a more holistic understanding of spinal health.

### 4.6. Limitations

This cross-sectional study cannot determine the temporal directionality between curvature and muscle degeneration; there is no determination of cause and effect. Also the supine kyphosis cannot be equated to the clinical kyphosis detected on standing X-rays. Muscle–fat% depends on accurate HU calibration and protocol; although we used soft-tissue clipping and a fixed attenuation threshold, variations in scanner model, reconstruction kernel, and patient body habitus may introduce bias. Potential confounders—such as age, body mass index, bone density, socio-economic status and physical activity—were not controlled for in this analysis; however, we were able to identify sex, spine surgery and compression fracture on the CT images to assess for confounding effects. While segmentation accuracy was high overall, small or thin muscles remain susceptible to partial-volume effects, e.g., trapezius which had no significant correlation. (See Figure 5 for the thresholding approach and Table 2 for segmentation performance context.) The utility is also limited in patients who are not ambulatory and have developed profound fatty atrophy of the paraspinal muscles. We attempted to eliminate this confounding effect by excluding patients with muscle–fat% > 50 but we did not have access to clinical records that could establish if these subjects were truly non-ambulatory. Although there were patients with minor scoliosis, patients with severe scoliosis or complex spine deformity were also not assessed.

### 4.7. Future Directions

Future work will include multi-center external segmentation benchmarking to evaluate generalizability across imaging protocols, harmonization of HU thresholds, and covariate-adjusted modeling to disentangle confounding factors. Future research should also perform head-to-head comparisons of the available methods of muscle–fat% quantitation using a range of thresholds and histopathology as the gold standard of reference to determine the best method for measuring intramuscular fatty atrophy in the setting of spinal deformity research. Longitudinal studies will be essential to determine whether baseline muscle–fat% predicts kyphotic progression or functional decline. Integration of muscle–fat% together with automated Cobb angle measurement could create a unified, fully self-contained spine-analysis framework. Ultimately, this approach could enable large-scale, opportunistic screening for hyperkyphosis in the supine position and musculoskeletal degeneration using existing CT archives.

## 5. Conclusions

We developed an automated CT-based pipeline for trunk muscle segmentation and muscle–fat% quantification across nine bilateral muscle groups. Greater thoracic supine kyphosis was consistently associated with higher muscle–fat%, with the strongest associations in the paraspinal and quadratus lumborum muscles. These findings were directionally consistent across both manual and automated cohorts and remained stable after confounder exclusion.

The pipeline achieved strong internal and external segmentation performance, supporting automated muscle–fat% as a reproducible and scalable biomarker that can be obtained opportunistically from routine CT. Although limited by the cross-sectional design and the use of supine CT rather than standing radiographs, these results support automated CT-derived muscle composition analysis as a practical complement to conventional curvature assessment and a potential tool for screening and longitudinal monitoring of hyperkyphotic deformity.

## Figures and Tables

**Table 1:** Software and hardware environment.

Component	Specification
Operating System	Linux (x86_64)
GPU	NVIDIA GeForce RTX 4090 (24 GB GDDR6X)
CUDA/cuDNN	12.8/9.8
Python/PyTorch	3.10.12/2.7.1+cu128
Core Libraries	nnU-Net v2.6.2, MONAI 1.5.1, NumPy 2.1.2, SciPy 1.15.3
I/O	NiBabel 5.3.2, SimpleITK 2.5.2, scikit-image 0.25.2
Visualization/Statistics	Matplotlib 3.10.7, pandas 2.3.3, scikit-learn 1.7.2

**Table 2:** Segmentation performance by muscle group across cohorts (CT). Dice coefficients are reported separately for the training, internal validation, and external segmentation benchmark cohort.

Muscle Group	Train Dice	Internal Dice	External Dice
Psoas	0.982	0.972	0.961
Quadratus lumborum	0.971	0.958	0.942
Paraspinal	0.985	0.974	0.963
Latissimus dorsi	0.962	0.948	0.932
Iliacus	0.976	0.964	0.949
Rhomboid	0.988	0.976	0.965
Trapezius	0.979	0.966	0.951
Rectus femoris	0.968	0.952	0.936
Vastus	0.973	0.959	0.944
Macro-average	0.976	0.963	0.949

**Table 3:** Intramuscular muscle–fat% (mean ± SD) by muscle across cohorts. AtlasDataset manual cohort with manual muscle segmentations (n=100), AtlasDataset automated cohort with nnU-Net segmentations (n=433), and the external segmentation benchmark cohort (TCIA CT-ORG subset, n=30) with manual muscle labels and automated segmentations.

Muscle	Manual Cohort (AtlasDataset, n=100)	Automated Cohort (AtlasDataset, n=433)	External Cohort (TCIA, n=30)
Psoas	5.7 ± 3.1	5.1 ± 3.1	7.0 ± 2.7
Quadratus lumborum	14 ± 6.9	14 ± 9.3	15 ± 5.8
Paraspinal	15 ± 9.2	15 ± 11	18 ± 7.2
Latissimus dorsi	19.04 ± 10.23	16.36 ± 9.29	19.02 ± 7.23
Iliacus	4.89 ± 2.19	4.48 ± 2.39	5.67 ± 3.32
Rhomboid	2.71 ± 1.81	2.24 ± 2.14	4.11 ± 2.54
Trapezius	2.78 ± 1.82	1.97 ± 1.71	2.40 ± 1.73
Rectus femoris	2.49 ± 2.54	2.07 ± 2.32	2.71 ± 7.01
Vastus	3.25 ± 2.17	2.52 ± 2.72	2.73 ± 2.61

**Table 4:** Correlation between thoracic supine kyphosis (Cobb angle) and muscle–fat% before and after confounder exclusion. Pearson correlation coefficients (*r*) with two-sided *p*-values (in parentheses) are reported for the AtlasDataset manual cohort (manual muscle segmentations) and the AtlasDataset automated cohort (nnU-Net segmentations). Baseline correlations are shown for the full cohorts (manual: n=100; model: n=433). Adjusted correlations exclude cases with prior spine surgery and profound fatty atrophy (paraspinal muscle–fat% >50%; manual: n=97; model: n=421).

Muscle	Manual Cohort (Raw)	Manual Cohort (Adjusted)	Automated Cohort (Raw)	Automated Cohort (Adjusted)
Cases studied, *n*	100	97	433	421
Psoas	0.17 (0.091)	0.21 (0.042)	0.19 (<0.001)	0.23 (<0.001)
Quadratus lumborum	0.23 (0.021)	0.28 (0.0063)	0.29 (<0.001)	0.33 (<0.001)
Paraspinal	0.30 (0.0022)	0.35 (0.0005)	0.38 (<0.001)	0.42 (<0.001)
Latissimus dorsi	0.18 (0.071)	0.21 (0.040)	0.16 (0.0008)	0.17 (0.0004)
Iliacus	0.11 (0.29)	0.16 (0.12)	0.18 (0.0002)	0.22 (<0.001)
Rhomboid	0.20 (0.048)	0.25 (0.015)	0.08 (0.093)	0.11 (0.023)
Trapezius	0.15 (0.13)	0.17 (0.089)	0.09 (0.078)	0.17 (0.0004)
Rectus femoris	0.16 (0.11)	0.19 (0.061)	0.13 (0.0058)	0.15 (0.0020)
Vastus	0.35 (0.0004)	0.38 (0.0001)	0.20 (<0.001)	0.32 (<0.001)

## Data Availability

Model codes and checkpoints as well as the Cobb angle measurements and muscle labels are available at Spinal-Muscle-Analysis (GitHub repository, https://github.com/onlineinfoh/Spinal-Muscle-Analysis (accessed on 26 February 2026)).

## Abbreviations

The following abbreviations are used in this manuscript:

3D	Three-dimensional
AI	Artificial Intelligence
CI	Confidence Interval
CT	Computed Tomography
CUDA	Compute Unified Device Architecture
cuDNN	CUDA Deep Neural Network library
Dice	Sørensen–Dice coefficient
EMA	Exponential Moving Average
GPU	Graphics Processing Unit
HU	Hounsfield Unit
ICC	Intraclass Correlation Coefficient
I/O	Input/Output
IQR	Interquartile Range
ITK-SNAP	Open-source 3D segmentation software
MONAI	Medical Open Network for AI
MRI	Magnetic Resonance Imaging
nnU-Net	Self-configuring deep learning segmentation framework
QA	Quality Assurance
QC	Quality Control
SD	Standard Deviation

## Appendix A

The following appendix material is provided in this appendix:

- Table A1. Case exclusion log for correlation analyses, detailing predefined confounder exclusions (prior spine surgery and profound fatty atrophy), quality-control removals, and muscle-specific exclusions.
- Table A2. Four-observer thoracic supine Cobb angle reliability in the AtlasDataset (n=533): per-rater mean ± SD (degrees), across-rater per-case SD summary, and inter-observer ICC(2,*k*) with 95% confidence interval.
- Table A3. Sex-stratified Pearson correlations between thoracic supine Cobb angle and muscle–fat% in the automated nnU-Net cohort, including male–female differences.
- Table A4. Compression fracture sensitivity analysis showing Pearson correlations with vertebral compression fractures included versus excluded.
- Table A5. Transitional vertebra sensitivity analysis showing Pearson correlations with transitional vertebrae included versus excluded.
- Figure A1. Sagittal midline CT reconstruction demonstrating fixed thoracic supine kyphosis due to ossification of the anterior longitudinal ligament.
- Figure A2. Manual-segmentation cohort (raw n=100; adjusted n=97): thoracic supine Cobb angle vs. muscle–fat% for all nine muscles shown as a 3×3 scatter-plot grid with Pearson’s *r* and two-sided *p*-values.
- Figure A3. Automated nnU-Net cohort (raw n=433; adjusted n=421): thoracic supine Cobb angle vs. muscle–fat% for all nine muscles shown as a 3×3 scatter-plot grid with Pearson’s *r* and two-sided *p*-values.

This appendix provides tables and figures accompanying the manuscript. It includes (i) detailed thoracic Cobb angle reliability summaries across four observers, (ii) the case exclusion log used for predefined confounder and quality-control sensitivity analyses, (iii) sex-stratified correlation results, and (iv) full scatter-plot grids for all nine muscles in both the manual-segmentation cohort and the automated nnU-Net cohort. External CT-ORG cases were used only for segmentation performance evaluation and are not included in Cobb angle–muscle–fat% correlation analyses.

**Table A1:** Case exclusion log for correlation analyses. Excluded cases for adjusted Pearson correlation analyses are listed by cohort. The main cohort rows report the exclusions applied in the adjusted analyses, and trapezius-only removals were applied only to trapezius correlations.

Cohort	Category	Case IDs
Manual cohort (manual masks)	Adjusted-analysis exclusions	101, 103, 155
Automated cohort (nnU-Net masks)	Adjusted-analysis exclusions	74, 94, 98, 237, 252, 288, 303, 387, 477, 480, 503, 531
Trapezius-only (both cohorts)	Trapezius-specific exclusions	76, 274, 275, 396

**Table A2:** Four-observer thoracic Cobb angle reliability in the AtlasDataset (n=533). Per-rater mean ± SD (degrees), across-rater per-case SD summary, and ICC(2,*k*) with 95% CI.

Measure	R1	R2	R3	R4	Overall
Mean ± SD (deg)	39.4 ± 13.9	38.3 ± 13.3	38.0 ± 12.8	38.6 ± 14.0	38.6
Across-rater SD (per-case), mean ± SD (deg)	—				3.4 ± 1.6
ICC(2,*k*) [95% CI]	—				0.980 [0.977, 0.982]

**Table A3:** Sex-stratified Pearson correlations between thoracic Cobb angle and muscle–fat% in the confounder-filtered automated nnU-Net cohort (male n=240 for most muscles, n=237 for trapezius; female n=181 for most muscles, n=180 for trapezius; total n=421 for most muscles and n=417 for trapezius *). Here, *r* denotes the Pearson correlation coefficient and *p* the corresponding two-sided p-value. The sex-specific sample sizes were sufficiently large to permit meaningful stratified assessment. Correlations remained positive in both males and females across all muscles and were broadly similar in magnitude across strata, with no qualitative change in the overall association pattern. These results support the interpretation that sex was not a major confounder of the association between thoracic Cobb angle and muscle–fat% after confounder removal.

Muscle	Male *r*	Male *p*	Female *r*	Female *p*
Psoas	0.27	<0.001	0.19	0.009
Quadratus lumborum	0.36	<0.001	0.30	<0.001
Paraspinal	0.42	<0.001	0.41	<0.001
Latissimus dorsi	0.19	0.003	0.13	0.092
Iliacus	0.25	<0.001	0.19	0.011
Rhomboid	0.16	0.014	0.05	0.500
Trapezius *	0.24	<0.001	0.08	0.270
Rectus femoris	0.18	0.004	0.10	0.160
Vastus	0.32	<0.001	0.31	<0.001

**Table A4:** Compression fracture sensitivity analysis showing Pearson correlations with vertebral compression fractures included versus excluded.

Muscle	Fractures Included	Fractures Excluded
Psoas	0.195 (<0.001)	0.187 (<0.001)
Quadratus lumborum	0.286 (<0.001)	0.262 (<0.001)
Paraspinal	0.384 (<0.001)	0.358 (<0.001)
Latissimus dorsi	0.161 (<0.001)	0.150 (0.003)
Iliacus	0.181 (<0.001)	0.201 (<0.001)
Rhomboid	0.081 (0.093)	0.085 (0.093)
Trapezius	0.085 (0.078)	0.156 (0.002)
Rectus femoris	0.133 (0.0058)	0.129 (0.011)
Vastus	0.196 (<0.001)	0.286 (<0.001)

**Table A5:** Transitional vertebra sensitivity analysis showing Pearson correlations with transitional vertebrae included versus excluded.

Muscle	Transitional Vertebrae Included	Transitional Vertebrae Excluded
Psoas	0.195 (<0.001)	0.193 (<0.001)
Quadratus lumborum	0.286 (<0.001)	0.282 (<0.001)
Paraspinal	0.384 (<0.001)	0.386 (<0.001)
Latissimus dorsi	0.161 (<0.001)	0.156 (0.002)
Iliacus	0.181 (<0.001)	0.183 (<0.001)
Rhomboid	0.081 (0.093)	0.083 (0.096)
Trapezius	0.085 (0.078)	0.139 (0.005)
Rectus femoris	0.133 (0.0058)	0.136 (0.006)
Vastus	0.196 (<0.001)	0.204 (<0.001)

### Sensitivity Analyses

Across all appendix analyses, the association between thoracic Cobb angle and muscle fat percentage remained directionally consistent and of comparable magnitude, supporting the robustness of the primary findings.

Sex-stratified analyses (Table A3) demonstrated overlapping effect sizes across males and females for all muscles, with no systematic reversal of correlation direction. While select muscles exhibited nominally stronger associations in females, full-cohort correlations consistently fell within the range defined by the sex-specific estimates, indicating that sex does not act as a major confounder of the observed relationships.

Sensitivity analyses restricted to cases with vertebral compression fractures (Table A4) and to cases with transitional vertebrae (Table A5) yielded correlation magnitudes comparable to those observed in the full cohorts. The preservation of association strength across these restricted subsets suggests that neither compression fractures nor transitional vertebrae materially influence the relationship between thoracic supine kyphosis and muscle fat infiltration.

Together, these findings indicate that the observed Cobb angle–muscle fat associations are stable across key anatomical and demographic strata and are unlikely to be driven by these predefined confounders.

## References

[1] Cobb J.R. (1948). Outline for the study of scoliosis. Instructional Course Lectures.

[2] Katzman W., Cawthon P., Hicks G.E., Vittinghoff E., Shepherd J., Cauley J.A., Harris T., Simonsick E.M., Strotmeyer E., Womack C. (2012). Association of spinal muscle composition and prevalence of hyperkyphosis: The Health ABC Study. J. Gerontol. A.

[3] Gstoettner M., Sekyra K., Walochnik N., Winter P., Wachter R., Bach C.M. (2007). Inter- and intraobserver reliability assessment of the Cobb angle: Manual versus digital measurement tools. Eur. Spine J..

[4] Dimar J.R., Carreon L.Y., Labelle H., Djurasovic M., Weidenbaum M., Brown C., Roussouly P. (2008). Intra- and inter-observer reliability of determining radiographic sagittal parameters of the spine and pelvis using a manual and a computer-assisted methods. Eur. Spine J..

[5] Wu W., Liang J., Du Y., Tan X., Xiang X., Wang W., Ru N., Le J. (2014). Reliability and reproducibility analysis of the Cobb angle and assessing sagittal plane by computer-assisted and manual measurement tools. BMC Musculoskelet. Disord..

[6] Beauchamp M., Labelle H., Grimard G., Stanciu C., Poitras B., Dansereau J. (1993). Diurnal variation of Cobb angle measurement in adolescent idiopathic scoliosis. Spine.

[7] Kado D.M., Christianson L., Palermo L., Smith-Bindman R., Cummings S.R., Greendale G.A. (2006). Comparing a supine radiologic versus standing clinical measurement of kyphosis in older women: The Fracture Intervention Trial. Spine.

[8] Wang L., Valencak T.G., Shan T. (2024). Fat infiltration in skeletal muscle: Influential triggers and regulatory mechanism. iScience.

[9] Aubrey J., Esfandiari N., Baracos V.E., Buteau F.A., Frenette J., Putman C.T., Mazurak V.C. (2014). Measurement of skeletal muscle radiation attenuation and basis of its biological variation. Acta Physiol..

[10] Crawford R.J., Filli L., Elliott J.M., Nanz D., Fischer M.A., Marcon M., Ulbrich E.J. (2016). Age- and level-dependence of fatty infiltration in lumbar paravertebral muscles of healthy volunteers. AJNR Am. J. Neuroradiol..

[11] Volpi E., Nazemi R., Fujita S. (2004). Muscle tissue changes with aging. Curr. Opin. Clin. Nutr. Metab. Care.

[12] Fortin M., Macedo L.G. (2013). Multifidus and paraspinal muscle group cross-sectional areas of patients with low back pain and control patients: A systematic review with a focus on blinding. Phys. Ther..

[13] Katzman W.B., Miller-Martinez D., Marshall L.M., Lane N.E., Kado D.M. (2014). Kyphosis and paraspinal muscle composition in older men: A cross-sectional study for the osteoporotic fractures in men (MrOS) research group. BMC Musculoskelet. Disord..

[14] Ding J.-Z., Kong C., Li X.-Y., Sun X.-Y., Lu S.-B., Zhao G.-G. (2022). Different degeneration patterns of paraspinal muscles in degenerative lumbar diseases: A MRI analysis of 154 patients. Eur. Spine J..

[15] Han G., Zhou S., Qiu W., Fan Z., Yue L., Li W., Wang W., Sun Z., Li W. (2023). Role of the paraspinal muscles in the sagittal imbalance cascade: The effects of their endurance and of their morphology on sagittal spinopelvic alignment. J. Bone Jt. Surg. Am..

[16] Zhang Y., Mandelli F., Mündermann A., Nüesch C., Kovacs B., Schären S., Netzer C. (2021). Association between fatty infiltration of paraspinal muscle, sagittal spinopelvic alignment and stenosis grade in patients with degenerative lumbar spinal stenosis. North Am. Spine Soc. J..

[17] Liao Y., Liu X., Xu T., Li C., Xiao Q., Zhang X. (2024). Association between paraspinal muscle fat infiltration and regional kyphosis angle in thoracolumbar fracture patients: A retrospective study. Sci. Rep..

[18] Isensee F., Jaeger P.F., Kohl S.A., Petersen J., Maier-Hein K.H. (2021). nnU-Net: A self-configuring method for deep learning-based biomedical image segmentation. Nat. Methods.

[19] Jaus A., Seibold C., Hermann K., Walter A., Giske K., Haubold J., Kleesiek J., Stiefelhagen R. *AtlasDataset* (GitHub Repository), 2023–2025. https://github.com/alexanderjaus/AtlasDataset.

[20] Jaus A., Seibold C., Hermann K., Walter A., Giske K., Haubold J., Kleesiek J., Stiefelhagen R. (2023). Towards Unifying Anatomy Segmentation. arXiv.

[21] Koo T.K., Li M.Y. (2016). A Guideline of Selecting and Reporting Intraclass Correlation Coefficients for Reliability Research. J. Chiropr. Med..

[22] Rister B., Shivakumar K., Nobashi T., Rubin D.L. (2019). CT-ORG: A Dataset of CT Volumes with Multiple Organ Segmentations (Version 1). Cancer Imaging Arch..

[23] Yushkevich P.A., Piven J., Hazlett H.C., Smith R.G., Ho S., Gee J.C., Gerig G. (2006). User-guided 3D active contour segmentation of anatomical structures: Significantly improved efficiency and reliability. NeuroImage.

[24] Yushkevich P.A., Gerig G. (2017). ITK-SNAP: An intractive medical image segmentation tool to meet the need for expert-guided segmentation of complex medical images. IEEE Pulse.

[25] Vallat R. (2018). Pingouin: Statistics in Python. J. Open Source Softw..

[26] Bland J.M., Altman D.G. (1986). Statistical methods for assessing agreement between two methods of clinical measurement. Lancet.

[27] Wesselink E.O., Pool J.J.M., Mollema J., Weber K.A., Elliott J.M., Coppieters M.W., Pool-Goudzwaard A.L. (2023). Is fatty infiltration in paraspinal muscles reversible with exercise in people with low back pain? A systematic review. Eur. Spine J..

